# Acetyl Groups in *Typha capensis*: Fate of Acetates during Organosolv and Ionosolv Pulping

**DOI:** 10.3390/polym10060619

**Published:** 2018-06-05

**Authors:** Idi Guga Audu, Nicolas Brosse, Heiko Winter, Anton Hoffmann, Martina Bremer, Steffen Fischer, Marie-Pierre Laborie

**Affiliations:** 1 Chair of Forest Biomaterials, University of Freiburg, Werthmannstr. 6, 79085 Freiburg im Breisgau, Germany; heiko.winter@biomat.uni-freiburg.de (H.W.); marie-pierre.laborie@biomat.uni-freiburg.de (M.-P.L.); 2 Laboratoire d'Étude et de Recherche sur le Matériau Bois LERMAB, Faculty of Science and Technology, University of Lorraine, Boulevard des Aiguillettes, BP 70239, 54506 Vandœuvre lès Nancy CEDEX, France; Nicolas.Brosse@univ-lorraine.fr; 3 Freiburg Materials Research Center (FMF), University of Freiburg, Stefan-Meier-Str. 21, 79104 Freiburg im Breisgau, Germany; 4 Institute of Plant and Wood Chemistry, Technische Universität Dresden, Pienner Straße 19, 01737 Tharandt, Germany; anton.hoffmann@forst.tu-dresden.de (A.H.); martina.bremer@forst.tu-dresden.de (M.B.); sfischer@forst.tu-dresden.de (S.F.)

**Keywords:** *Typha capensis*, native acetate, lignin, MWL, ionic liquid lignin, ethanol organosolv lignin

## Abstract

During biomass fractionation, any native acetylation of lignin and heteropolysaccharide may affect the process and the resulting lignin structure. In this study, *Typha capensis* (TC) and its lignin isolated by milling (MWL), ionosolv (ILL) and organosolv (EOL) methods were investigated for acetyl group content using FT-Raman, ^1^H NMR, 2D-NMR, back-titration, and Zemplén transesterification analytical methods. The study revealed that TC is a highly acetylated grass; extractive free TC (TC_extr_) and TC MWL exhibited similar values of acetyl content: 6 wt % and 8 wt % by Zemplén transesterification, respectively, and 11 wt % by back-titration. In contrast, lignin extracted from organosolv and [EMIm][OAc] pulping lost 80% of the original acetyl groups. With a high acetyl content in the natural state, TC could be an interesting raw material in biorefinery in which acetic acid could become an important by-product.

## 1. Introduction

Grass lignins have the distinct feature of containing significant amounts of p-coumaric and ferulic acids [1,2,3], and acylation mainly bound to the γ-carbon of S units [2,3,4]. In kenaf, sisal and abaca, 45–80% of alkyl-aryl ether-linked S units are reported to carry an acetyl group [4]. During biomass fractionation, acetylation is likely to play a significant role due to the ease of acetate cleavage [5]. In turn, the release of acetic acid during biorefining might impair control of the pH [5,6], while providing opportunities for acetic acid recovery [6]. Although acetyl groups were detected in the cell wall of *Miscanthus x giganteus*, however, after pulping with 1-butylimidazolium hydrogen sulfate, acetyl groups were not detected in the isolated lignin [7]. In the case of wood pulping with dialkyl imidazolium salts such as 1-ethyl-3-methylimidazolium acetate ([EMIm][OAc]), acetyl transfer was revealed as an important side-reaction [8,9,10]. [EMIm][OAc] swells lignocellulose effectively, eventually causing derivatization of its structural polymers [11,12,13]. In particular, xylan deacetylation and lignin acetylation have been observed [10]. In [EMIm][OAc], imidazole, a degradation product of imidazolium, plays the role of acetyl transfer onto cellulose [8], a side-reaction catalyzed by lignin [9]. Although the source of the acetic acid is not known, acetate cleavage in lignocellulose and the presence of acetates in [EMIm][OAc] are both potential sources of acetic acid for this side-reaction [9]. When pulping a grass with [EMIm][OAc], any native acetylation of lignin (and of heteropolysaccharides) will thus likely affect the process and resulting lignin structure.

*Typha capensis* (TC) is a prolific invasive grass native of Southern Africa, which has high resistance to drought [14] and high annual productivity (57 t/ha dry matter) [15]. However, its use is marginal, principally in medicinal treatments, fabrication of hand brooms and woven mats and in roofing [14]. Therefore, TC has a strong potential as feedstock for bioenergy, bioproducts and bio-based chemicals without competing with food applications. In recent investigations of TC, we reported that native TC comprised of ca. 18% extractives, 39% cellulose, 19% hemicellulose and 23% lignin [16]. Furthermore, it was shown that TC grass can be effectively fractionated with organosolv and [EMIm][OAc] pulping, releasing distinct lignin residues [16]. While FTIR of TC MWL revealed acetylation, acetate bands were not detected in EOL and ILL, perhaps due to method-sensitivity limits [16]. Therefore, to provide a thorough view of the extent of acetylation in native TC and monitor potential acetate transfer during TC pulping, more sensitive analytical methods are required. To the best of our knowledge, the native state and fate of possible acetyl groups in TC grass during fractionation have not been reported.

A common method for determining native acetate content in lignin is modified derivatization followed by reductive cleavage (DFRC), which selectively cleaves α- and β-O-4 linked lignin acetates [17,18,19]. The specificity of the method to α- and β-O-4 linked lignin acetates and method limitations associated with the use of tetracosane or 4,4-ethylidenebisphenol as internal standard (IS) do not allow a comprehensive view of total acetylation content [20]. Other methods hinging on ester cleavage and quantification of the thereby released acetic acid [21,22] cannot distinguish covalently bound acetate from free acetic acid [6]. This is particularly problematic in procedures that involves acetylating agents to temporarily protect hydroxyl groups acetates in which the acetylating agents and their hydrolysis products may be present in adsorbed form [6,8]. Thus, there remains a need for a sensitive and selective method to accurately quantify covalently bound acetates in biomass and monitor their fate during pulping. Although not widely applied, the Zemplén method recently revisited by Zweckmair et al. [6], could be a valuable approach. The method entails transesterification of covalently bound acetyl groups by catalytic action of anhydrous sodium methanolate in excess of methanol to form methyl acetates, which are thereafter quantified by gas chromatography [6]. Following introduction of the method to non-soluble polysaccharides by Zweckmair et al. [6], the Zemplén method has been successfully applied to monitor polysaccharides’ acetylation during wood pulping with 1,3-dialkylimidazolium acetate [8]. To the best of our knowledge, application of this method to lignin acetylation has not been reported to date.

The aim of this study is to investigate the native acetylation state of *Typha capensis* and examine its fate during pulping with [EMIm][OAc] and with ethanol. We further assess the value of the Zemplén transesterification method to quantify covalently bound acetates to lignin and TC biomass [6,8]. Coupling the Zemplén method with FT-Raman vibrational spectroscopy, back-titration, ^1^H as well as 2D NMR, a comprehensive view of TC acetylation state and fate is provided.

## 2. Materials and Methods

### 2.1. Materials and Sample Preparation

Chemicals of analytical grade were purchased from Merck Germany and used as supplied, including maleic acid, DMSO-*d*_6_, sodium hydroxide, 1-ethyl-3-methylimidazolium acetate, anhydrous methanol, acetic acid, methyl acetate, sodium methanolate, vanillin, sulfuric acid (97%), hydrochloric acid (37%), ethanol, dichloromethane, ethyl acetate, cellulose acetate (with acetyl value of 39.3–40.3 wt %), and acetyl chloride–^13^C_2_ (99 atom % ^13^C). Beech wood was sampled from southern Germany forests. Acetylated beech wood and coconut trunk were kindly supplied by Rhodia Acetow/Accoya (Freiburg) and the University of Hamburg, Germany, respectively.

*Typha capensis* sample was uprooted, cut to ≤2 cm pieces, and sundried. The protocol edited by Hames et al. [23] was used for sample preparation as previously described [16]. Briefly, the cut raw TC was oven dried at 40 °C for 48 h, milled to obtain particle size ≤ 0.4 mm. The milled raw TC was sequentially and exhaustively extracted using a Soxhlet Extraction apparatus with water, ethanol and dichloromethane (DCM), refluxed for 16, 16 and 8 h, respectively, to obtain extractive free TC (TC_extr_) [23], and kept until further use.

### 2.2. MWL Isolation

The procedure by Bjorkman as modified by Obst and Kirk and Rencoret et al. was used to process MWL from TC_extr_ [24,25].

### 2.3. Ionic Liquid Mediated Lignin Extraction

The procedure published by Sun et al. [26] was used with slight modifications as described previously [16]. Briefly, TC_extr_ was weighed in a conical flask and 1-ethyl-3-methylimidazolium acetate was added to obtain a biomass to solvent ratio of 1:20 (*w*/*w*). This was followed by reacting at 110 °C in oil bath with magnetic stirring for 16 h. At the end of the reaction, the content was cooled to room temperature using ice water. About 7.5 mL of acetone/water (1:1, *v*/*v*) per g of ionic liquid used was added and briskly stirred. The dissolved lignin in solution with the ionic liquid and acetone/water was separated from the cellulose-rich residue by filtration through Whatman filter paper number 4 using Buchner funnel under reduced pressure. The residue was washed 3 times using acetone/water solution and filtered again. All the filtrates were joined together, and acetone was evaporated either by rotary evaporator or by magnetic stirring overnight in an open beaker under the fume hood. The lignin in the liquid fraction was collected by centrifugation at 4000 rpm for 20 min. The lignin was lyophilized and then oven dried overnight at 40 °C. In order to reuse the IL, water in the liquid fraction was evaporated to recover IL under reduced pressure. A purification step was performed on the lignin by Soxhlet extraction using ethanol, ethyl acetate and n-hexane refluxed for 8 h sequentially [20]. However, washing with 0.1 M HCl proved more effective in obtaining relatively pure ILL.

### 2.4. Sulfuric Acid Catalyzed Ethanol Organosolv Lignin Extraction

The procedure by El Hage et al. [27] was used with slight modification as described previously [16]. Briefly, TC_extr_ was first autohydrolyzed. The dried autohydrolyzed sample was weighed and loaded into the Parr reactor and ethanol/water solution—65:35 (*v*/*v*) containing 0.5% sulfuric acid (*w*/*w*) was added to obtain a solid to liquid ratio of 1:9 and reacted at 170 °C for 1 h. This was followed by cooling and filtration using Whatman filter paper number 4 to separate liquid from solid phases. The residue was washed three times using warm (60 °C) ethanol/water (4:1 ratio, *v*/*v*) at a volume of about 2 mL per gram of pretreated sample. The filtrates were combined, and deionized water added. The mixture was cooled overnight in a refrigerator at 4 °C and centrifuged at 4000 rpm for 20 min to precipitate the ethanol organosolv lignin (EOL). The recovered EOL was further washed with deionized water and oven dried at 40 °C for about 12 h.

### 2.5. Raman Vibrational Spectroscopy of Native TC

Information on unprocessed or native TC was obtained from a cut and dried TC sample. Raman spectrum of the sample was collected using Bruker MultiRam spectrometer with Ge diode as detector that is cooled with liquid nitrogen. A CW-Nd:YAG-laser with an exciting line of 1064 nm was applied as a source of light for the excitation of Raman scattering. Spectra were recorded over a range of 3500–400 cm^−1^ with 200 scans using spectral resolution of 3 cm^−1^ and laser power of 100 mW. The operating spectroscopy software Opus v. 7.2 (Bruker, Billerica, MA, USA) was used to acquire the data, locate peaks positions and process the spectral data.

### 2.6. ^1^H and 2D NMR (HSQC)

^1^H NMR was performed for the lignin isolates. 18 mg of each sample and 9 mg maleic acid as IS were dissolved in 0.6 mL DMSO-*d*_6_. The spectra were acquired on a Bruker Avance-400 NMR spectrometer, the number of scans was 32 and interscan delay time was 1 s, and ~1 s acquisition time. The aromatic and aliphatic acetates were estimated with reference to the IS according to Equation (1).

(1) $$c_{acetyl}=\frac{n_{maleic\;acid}}{\frac{\int_{6.142\;\mathrm{ppm}}^{6.313\;\mathrm{ppm}}{\left(-CH\right)}_{2}}{2}}\times \;\frac{\int_{1.810\;\mathrm{ppm}}^{2.091\;\mathrm{ppm}}\left(-CH_{3}\right)+\int_{2.169\;\mathrm{ppm}}^{2.312\;\mathrm{ppm}}\left(-CH_{3}\right)}{3}\times \frac{1}{m_{sample}}\;\left(\frac{\mathrm{mmol}}{g}\right)$$

where $c_{acetyl}\left(\frac{\mathrm{mmol}}{g}\right)$ is the content of acetyl groups in the sample, $n_{maleic\;acid}\left(\mathrm{mmol}\right)$ is the amount of IS and $m_{sample}\left(g\right)$ is the mass of the analyzed lignin sample. The peak area of maleic acid can be attributed to two protons and the peak areas of aromatic and aliphatic acetyl groups can be attributed to three protons.

TC whole cell wall analysis was performed on 100 mg ball milled and dried TC_extr_ samples, swollen in 0.75 mL DMSO-*d*_6_. 2D NMR HSQC data were acquired on a Bruker Avance-400 NMR spectrometer at 300 K based on the method described by Lu and Ralph [28], as modified by Rencoret et al. [29]. For the lignin samples, 40 mg sample was dissolved in DMSO-*d*_6_ and HSQC data acquired as described above. Data was analyzed using Bruker TopSpin 3.2 software. Volume integrals were performed to estimate C-H correlations associated with acetate using Equation (2). The G2 correlation was used as a reference in which the integral of G2 was made to be equal to 1.

(2) $$\%Acetate=\;\frac{\int_{ppm}^{ppm}\left(ia+iia+iiia\ldots na\right)}{\int_{ppm}^{ppm}\left(1+2+3+\cdots n+ia+iia+\cdots .na\right)}\times 100\%$$

where *ia*, *iia*, … *na* are integrals of correlations associated with acetyl groups, while 1, 2, … *n* are integrals of all correlations assigned in Table S1 (Supplementary materials). Note that unknown (unassigned) correlations were not integrated.

### 2.7. Zemplén Transesterification Reaction for Degree of Acetylation Determination and Back-Titration Methods

Sample preparation and GC/MS analysis were based on the procedure by Zweckmair et al. [6] with slight modification. A six-point calibration was done with known concentrations of acetylsalicylic acid. The method was first optimized to ensure that the peak areas of the samples analyzed were within the range of the peak areas of the IS (^13^C_2_-labelled acetyl vanillin) such that the response factor of the IS be of the same order of magnitude as that of the compounds analyzed [30]. Furthermore, based on the concentrations of acetylsalicylic acid used for the calibration curve, and for knowledge of the expected acetyl content, quantity of the samples for analysis were optimized to fit within the calibration curve limits. Stock solution 1 was prepared by dissolving 10 mg of ^13^C_2_-labelled acetyl vanillin as IS in 4 mL of anhydrous methanol. Then, between 0.150 to 2 mg of each sample was transferred into a vial. Specific ranges applied were 0.150 to 0.350 mg for cellulose acetate and ethyl acetate, 0.25 to 0.4 mg for acetylated Beech wood and about 2 mg for lignins and grass/wood biomass. 50 µL anhydrous Methanol, 100 µL standard solution (Stock 1), 1000 µL sodium methanolate (0.5 M in MeOH) were added into each sample and the vials sealed with 1.3 mm silicon/PTFE septa crimp caps and incubated at 35 °C for 20 min. GC/MS analysis was thereafter conducted on CAR/PDMS (= Carboxen/Polydimethylsiloxane (PDMS) with 85 µm film thickness from Supelco (Bellefonte, PA, USA) using column (Type: ZB-5MS, Manufacturer: Phenomenex), dimensions 30 m × 0.25 mm i.d. × 0.25 µm film thickness, programmed as follows: constant column flow—0.9 m/min; gas carrier—helium; temperature gradient profile—heated to 30 °C in 1 min, then up to 50 °C in 8 min and up to 200 °C in 10 min. Data was acquired in SIM mode selecting 74 *m*/*z* and 76 *m*/*z* for detection of methyl acetate analyte and ^13^C_2_-labelled derivative, respectively, at 120 s dwell time each, Zweckmair et al. [6].

For the back-titration method, the procedure reported by Kim et al. [31] was used. Briefly, all samples, except ethyl acetate, were first dried at 105 °C for 2 h and 500 mg weighed in a 250-mL round bottom flask. 40 mL of 75% ethanol was added, closed and sealed with parafilm and reacted at 60 °C for 30 min. 40 mL of 0.25 M NaOH was accurately added and further reacted at 60 °C for 15 min. The flasks were withdrawn and kept at room temperature to settle for 48 h, followed by back-titration with 0.25 M HCl using phenolphthalein as indicator. Acetyl group was calculated based on the released OH following NaOH reaction that was thereafter consumed by HCl.

The two methods were validated by running tests using cellulose acetate and ethyl acetate whose acetyl content are known. The acetyl contents of other monocotyledons and dicotyledon whose acetyl contents have been reported from literature, such as *Miscanthus x giganteus*, coconut trunk, beech wood, and acetylated beech wood were also tested to further verify the methods.

## 3. Results

### 3.1. Structural Analysis of Native TC by Raman Spectroscopy

**Figure 1:**
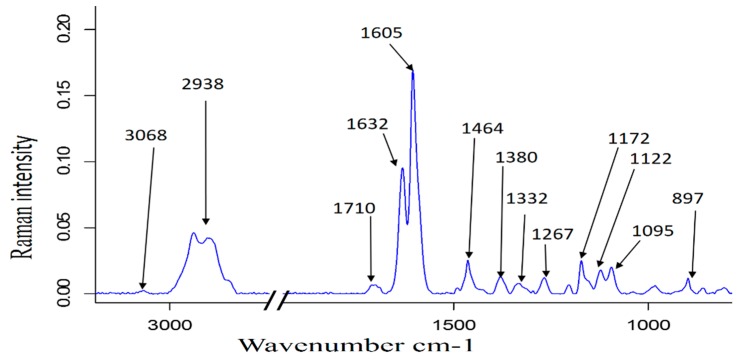
Raman spectrum of native *Typha capensis*.

The Raman spectrum of native TC (Figure 1) shows the presence of both carbohydrates and lignin, based on established band assignments) [32,33,34,35,36], Table S1 (Supplementary Materials). The intense and broad band around 2938 cm^−1^ and broad band at 1710 cm^−1^ might stem from acetyl groups in lignin (Table S1, Supplementary Materials) [32,33,34,35,36], hemicelluloses, and/or from residual protein, which was present in ca 4.8% as determined by nitrogen content from elemental analysis [16]. High Raman band intensities were observed for lignin aromatic skeletal vibrations at 1605 and the band at 1632 cm^−1^ that can be assigned to coniferaldehyde and sinapaldehyde [33]. Contributing to the band at 1632 cm^−1^ are cinnamic acid esters, which are known to be contained in herbaceous plants [33]. Duplet of band around 1605 and 1632 cm^−1^ is typical for herbaceous plants lignins that contain substantial proportion of H in addition to S and G structures.

### 3.2. Isolated Lignin Analysis by ^1^H NMR

**Figure 2:**
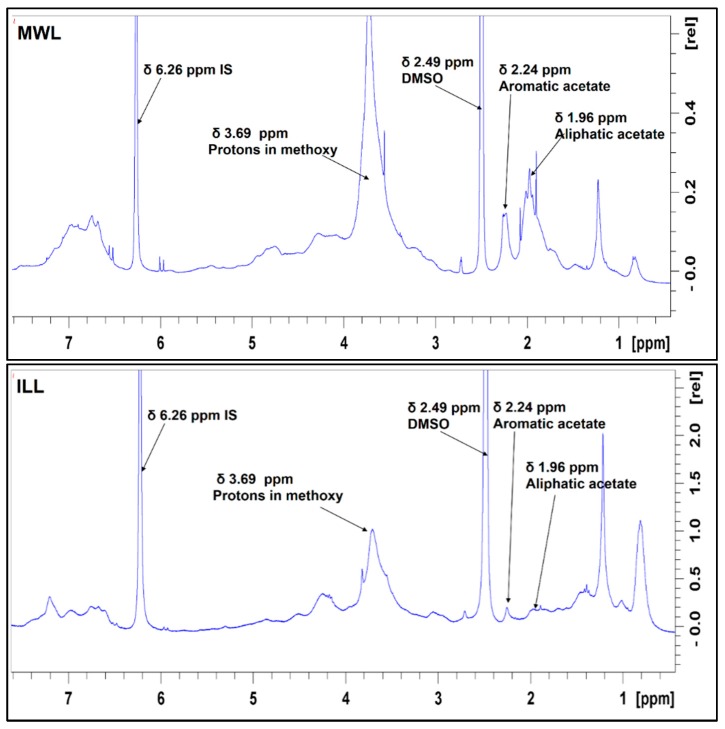

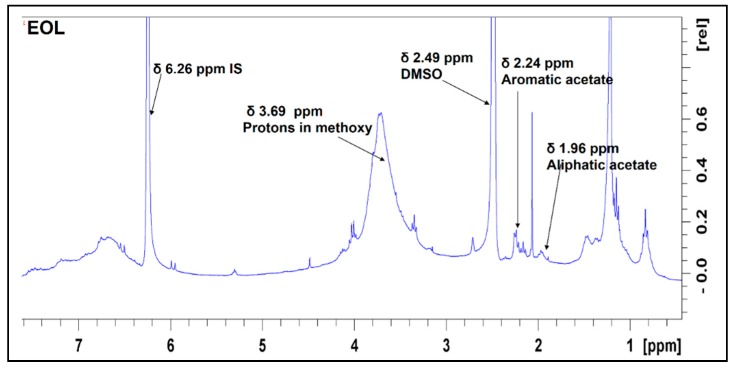
^1^H NMR spectra of lignin isolates (MWL, ILL and EOL) revealing acetate of varying intensity.

^1^H NMR spectra for the three lignins (Figure 2) reveal both aliphatic and aromatic acetates at 1.96 and 2.24 ppm in the spectra of the MWL, ILL and EOL lignins, [37,38]. Integration of resonances allowed estimation (Equation (1), Figure S1, Supplementary Materials) of the total acetyl associated units as 1.63 mmol/g for MWL, 0.76 mmol/g for ILL and 0.68 mmol/g for EOL, suggesting acetyl cleavage during ionic liquid and organosolv pulping. However, the interscan delay of 1 s used in acquiring the proton NMR data may not allow complete relaxation of the spin systems; therefore, this quantification with the proton NMR data must be taken with care.

### 3.3. Isolated Lignin and TC_extr_ Analysis by 2D HSQC

**Figure 3:**
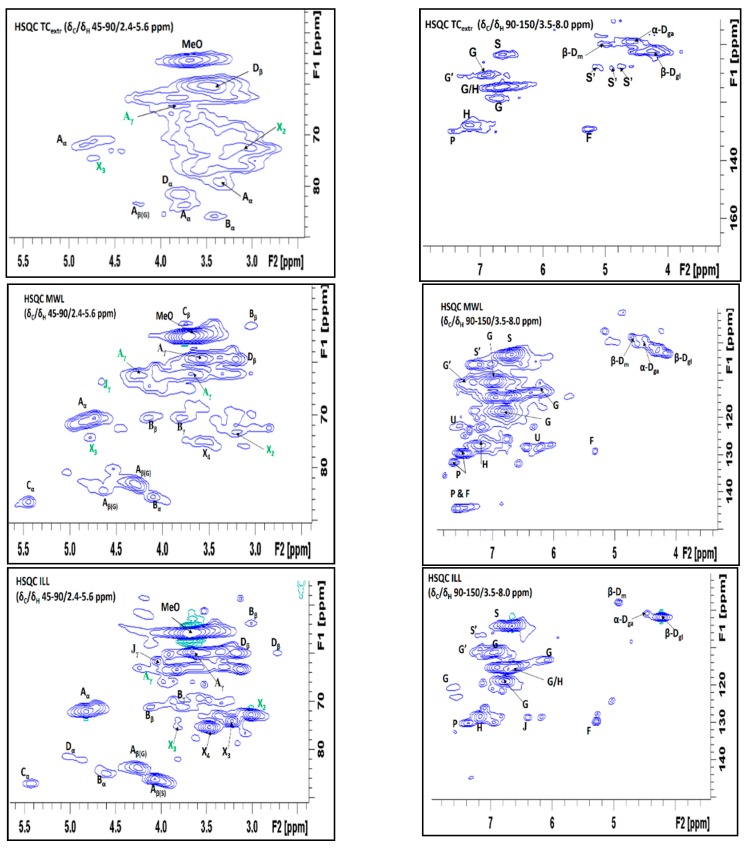

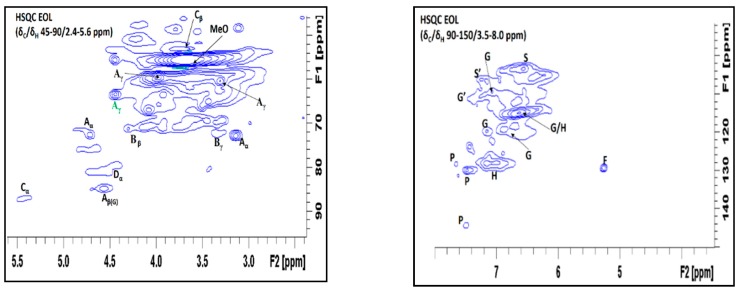
HSQC Spectra showing side chain and aromatic/unsaturated regions for TC_extr_ and TC lignin isolates (MWL, ILL and MWL), assignment in Table S2 (Supplementary Materials). Key: Symbols for acetylated units are in green highlight; β-O-4 alkyl-aryl ethers, including C_γ_-H_γ_ in γ-acetylated β-O-4′ substructures (**A**); Resinols (**B**); Phenylcoumarans (**C**); Spirodienone (**D**); Cinnamyl alcohol end groups, include C_γ_-H_γ_ in cinnamyl acetate end groups (J); *p*-coumaric and ferulic acids (P&F); β-d-Mannosyl (mannose residues) (β-D_m_); α-d-Galactosyl (galactose residues) (α-D_ga_); β-d-Glucosyl (Glucose residues) (β-D); C2−H2 in 2-O-acetyl-β-d-xylopyranoside (X2); C3−H3 in β-d-xylopyranoside (X3); C4−H4 in β-d-xylopyranoside (X4); Unknown (U).

The lignin isolates and TC_extr_ were further characterized by 2D HSQC (Figure 3). ^13^C and ^1^H correlations were assigned based on prior publications [2,18,29,39,40,41,42,43], Table S2 (Supplementary Materials). In previous works, it has been shown that HSQC NMR is a powerful method for the characterization of the acetylated moieties in lignin [43]. In the non-oxygenated aliphatic region, a useful hint is the prominent methyl in acetate peak linked to xylan moieties centered at δ_C_/δ_H_ 20.8/2.0 ppm correlations [43], (region is not shown). In the aliphatic oxygenated region, the spectra exhibit correlations (~δ_C_/δ_H_ 61.9–63.2/3.6–4.3) corresponding to the β-O-4′ linkages with acetylated γ-carbon of lignin units as previously described by del Rio et al. [2,18]. The MWL further exhibit signals at δ_C_/δ_H_ 64.1/4.71 ppm due to C_γ_-H_γ_ in cinnamyl acetate end groups. The complete absence of α-acylated β-O-4′ substructures, which should appear in all the samples at correlation δC/δH 75/6.1 ppm, confirms the fact that the aliphatic lignin side chain of TC, like most herbaceous lignins, is acetylated exclusively at the gamma-carbon [2].

In TC_extr_, MWL and ILL, correlations associated with polysaccharides were observed, assigned to anomeric β-d-glucosyl (δ_C_/δ_H_ 102.0/4.24 ppm), β-d-mannosyl (δ_C_/δ_H_ 98.4/4.84 ppm) and α-d-galactosyl (δ_C_/δ_H_ 99.4/4.45 ppm) units. Interestingly, acetate groups linked to polysaccharides were detected at δ_C_/δ_H_ 73.2/2.87 and 74.5/4.73 ppm, and assigned to C_2_-H_2_ correlations in 2-*O*-acetyl-β-d-xylopyranoside and C_3_-H_3_ in 3-*O*-acetyl-β-d-xylopyranoside, respectively. For EOL, the low concentration of sugars in the spectra suggests an extensive hydrolysis of polysaccharides under the acidic conditions used as revealed earlier by sugar analysis [16].

Acetylation content of the lignin side chain and of the sugars was tentatively computed from the HSQC spectra by integration of the signals (Equation (2)). HSQC full spectra and volume integration data presented at as Figure S2 and Table S3 (Supplementary Materials). The values are % of total integrals of all the signals for each sample. Caution on interpretation of these data is necessary since incomplete signal relaxation lead to underestimation for H and *p*-coumarate (PCA) [39,44]. The G_2_ correlations have been found to be the most stable signals per mg lignin [7]; this was therefore used as a reference peak in the volume integration. Accordingly, the calculated acetyl values are only indicative. Taking this into account, the TC_extr_ and MWL exhibit higher acetylation degrees (13% and 28%, respectively), whereas ILL and especially EOL display lower acetylation degree (10% and 2%, respectively). The acetyl signals linked to lignin and hemicelluloses are distinct from each other in the HSQC; therefore, the portion of acetyl groups for each can be roughly estimated from their volume integration. In all samples, more than half of the total integrated volume of acetyl signals are connected to lignin, with EOL having the highest relative share (TC_extr_: ~60%, MWL: ~70%, ILL: ~60%, EOL: ~80%). Correspondingly, the MWL and ILL have the highest proportions of acetyl groups connected to xylan (TC_extr_: ~40%, MWL: ~30%, ILL: ~40%, EOL: ~20%).

### 3.4. Degree of Acetylation by Zemplén Transesterification Reaction and by Back-Titration Method

The absolute contents of acetyl groups as determined by Zemplén transesterification and back-titration are compared (Table 1). According to both methods, the MWL presents the highest acetyl content closely followed by TC_extr_. Both methods clearly reveal lower acetyl content for EOL and ILL. In fact, by reference to the native material, more than 80% of the original acetyl groups appear to be lost during ILL and EOL processing.

## 4. Discussion

### 4.1. Comparison of Acetyl Group Values by Different Methods

**Figure 4:**
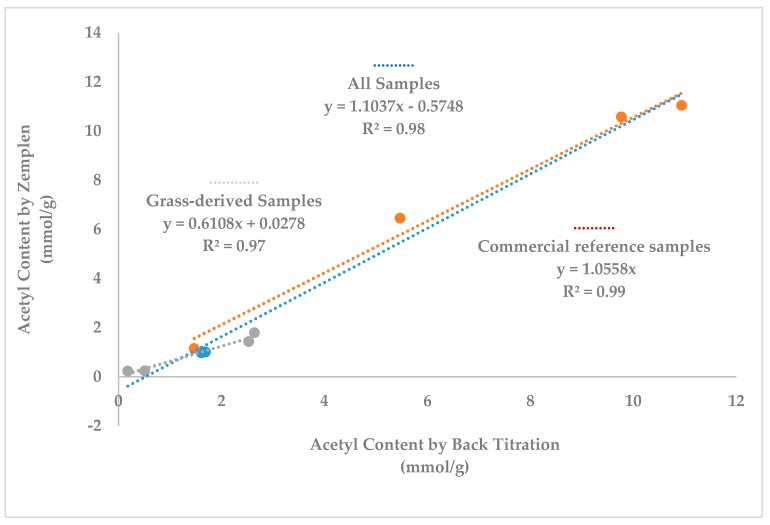
Corrrelations between acetyl contents measured by Zemplén and back titration methods validating the accuracy and sensitivity of the Zemplén method for acetate analysis in lignocellulosics.

Each quantitative method applied for acetyl determination resulted in similar trends in acetyl content; the MWL and native TC (TC_extr_) have the highest acetyl values whereas ILL, EOL as well as the cellulose rich residue from ionic liquid treatment have the lowest acetyl values (Table 1). Gratifyingly, the acetyl content estimates from the Zemplén method strongly correlates to that measured with titration methods (R^2^ = 0.98). In addition, when one pools data from all grass-derived biomasses on the one hand and all commercial reference samples on the other hand, distinct linear trends are found (R^2^ = 0.97 and 0.99, respectively) (Figure 4). Overall, these correlations confirm the value of the Zemplén method as a relative measure of acetyl content within a given type of biomass. Differences in correlation might stem from differences in molecular weights, chemical structure, and overall solubility of the materials.

For example, with all methods, the EOL had significantly lower value compared to ILL. However, our data from Zemplén show that EOL and ILL is almost equal. Checking the solubility (Plate S1, Supplementary Materials), EOL looks more dissolved. This suggests that almost all the bound acetyl group was released in EOL, while in the ILL and MWL that are not fully dissolved, the acetyl groups may not have been released completely. Although Zweckmair et al. [6] proposed that the solubility of the sample does not affect the outcome for cellulose, our experience suggests it might when comparing different substrates. This is subject to further study. On the other hand, back-titration methods likely overestimate acetyl content in chemically modified biomass especially, since residual adsorbed acetic acid present after chemical modification cannot be deciphered with the back-titration approach (Zweckmair et al.) [6]. Overall, the Zemplén method for acetyl content determination of lignocellulosics is thus confirmed as a valid and sensitive technique, allowing to determine covalently bound acetate in biomass. The small sample requirement by the Zemplén method could be an advantage, especially where sample limitation is an issue.

### 4.2. Acetyl Groups in TC_extr_ and MWL

The acetyl values detected for TC_extr_ are clearly higher compared to wood where acetyl contents in the range of 1 to 5 wt % were detected [45,46,47]. Additionally, acetyl values up to 4.8 wt % were determined for various monocotyledons using different analytical methods [48,49,50,51,52,53,54,55,56,57,58,59,60,61]. The highest acetyl values for monocotyledons found in literature were 6.2–7.0 wt % for oil palm trunk fractions determined by solid-state ^13^C NMR [62]. Values of the validation data conducted in this study for Beech wood, *Miscanthus x giganteus* and Coconut trunk conform to values reported from literature. The absolute value of 11 wt % that is representative of the total acetyl content in TC grass by back-titration (Table 1) reveals that TC_extr_ exhibits higher acetyl content in comparison to the values for other lignocelluloses reported earlier based on our thorough literature research [45,46,47,48,49,50,51,52,53,54,55,56,57,58,59,60,61,62,63,64].

With 11 wt % by back-titration method and 8 wt % by Zemplén, the acetyl values for *Typha capensis* MWL are also in a high range. In an earlier study, 0.82 mmol/g lignin was obtained from Kenaf MWL, corresponding to 9 wt % acetyl in the detected G and S units yielded by the applied modified DFRC method [4]. As the DFRC method only comprises lignin units connected by ether bonds, the values aren’t necessarily representative of the whole lignin.

### 4.3. Changes during EOL Pulping

The EOL was generally observed to have the lowest acetyl content compared to the MWL and the ILL (Table 1). In the HSQC, the absence of acetyl group related correlations associated with β-d-xylopyranoside in the EOL reflects the removal of hemicellulose by organosolv treatment. Acetate in the EOL is predominantly on lignin because the hemicelluloses have been hydrolyzed [27]. The fact that a part of the acetyl groups was not cleaved during the EOL production is not uncommon. After steaming of birch wood and wheat straw, acetyl contents of 7.8 wt % and 3.0 wt %, respectively, were detected on xylan precipitated from the liquid fraction [65]. Compared to examples from literature on alkali-extracted and kraft pulping [65,66], the low detected acetyl content of EOL suggests that most of the acetyl groups present in TCextr have been removed during the organosolv treatment.

### 4.4. Changes during [EMIm][OAc] Pulping

Acetyl values of the *Typha capensis* ILL are consistently slightly higher compared to the EOL but clearly lower compared to the MWL (Table 1), indicating that there were losses of acetyl groups during ionic liquid treatment. Based on a two-stage sulfuric acid hydrolysis and sugar analysis of the hydrolysate that followed using high performance anion-exchange chromatography with pulsed amperometric detection (HPAE-PAD), the residue from [EMIm][OAc] liquid treatment consists mainly of cellulose (70 wt % glucan, 10 wt % xylose and 14 wt % lignin). Analysis of this cellulose-rich residue from [EMIm][OAc] treatment results in acetyl value of 3 wt % (back-titration). This might arise from the inherent xylan and/or lignin composition of the residue or from an acetylation reaction occurring during pulping as previously observed with [EMIm][OAc] treatment and its thermal degradation product 1-acetylimidazol [8,9]. It appears that some of the native TC acetyl groups, value (2.53 ± 0.06 mmol/g), have partitioned between lignin (0.51 ± 0.01 mmol/g), cellulose rich pulp (0.7 ± 0.02 mmol/g) and other fractions cleaved during the process. In TC_extr_ and ILL, the acetate signals connected to lignin and hemicellulose moieties obtained by integrated volume of acetyl signals of the HSQC method shows similar signal distribution—~60% connected to lignin and ~40% connected to hemicellulose moieties. A previous study [16] showed that TC_extr_ and ILL are composed of 27 wt % and 7 wt % of non-cellulosic sugars, respectively. Even though HSQC values are indicative, this suggests that ILL has more acetyl groups per sugar unit in comparison to TC_extr_. Apparently, the hemicellulose part of ILL in comparison to TC_extr_ is enriched in acetyl groups, either by acetylation of hemicelluloses and/or privileged conservation of acetylated hemicellulose fractions during [EMIm][OAc] treatment. The observation that cellulose rich residue has been acetylated during [EMIm][OAc] treatment, together with the previously observed cellulose acetylation by this ionic liquid [8,9] suggests rather additional acetylation than preferential conservation of acetylated hemicellulose fraction during ionic liquid treatment of *Typha capensis*. Based on these findings, it is likely that during [EMIm][OAc] treatment, part of the acetyl groups in *Typha capensis* was relocated from lignin to cellulose and hemicelluloses, while another part of lignin acetyl groups was released during ionic liquid treatment.

## 5. Conclusions

To the best of our knowledge, the Zemplén transesterification method is demonstrated for the first time to accurately monitor the fate of lignocellulose acetyl groups during pulping. The Zemplén method correlates well with the back-titration method and also reveals similar trends than those hinted by NMR and Raman spectroscopies. The Zemplén method is advantageous due to its high sensitivity and small sample requirement. While the back-titration method quantifies total acetyl content in TC grass, the Zemplén method discriminates for covalently bound acetyl groups and is therefore particularly sensitive for grasses and their pulping products. TC exhibits higher acetyl content than other lignocellulosic materials reported in literature. The HSQC further indicated that the acetyl groups are linked on both lignin and hemicelluloses moieties with acetate in lignin exclusively connected through the γ-side chain. Organosolv and [EMim][OAc] pulping remove the majority of acetyl groups in lignin compared to MWL. Several dynamics occurred during [EMIm][OAc] treatment in which deacetylation and translocation of acetate from lignin to hemicelluloses moieties as well as to a cellulose-rich residue were observed.

## Figures and Tables

**Table 1:** Acetyl group contents of *Typha capensis* based samples and some reference samples determined by Zemplén transesterification reaction in comparison with back-titration values.

Acetyl Content				
Sample	Zemplén		Back-Titration	
(−)	(mmol/g)	(wt %)	(mmol/g)	(wt %)
*Typha capensis* samples				
TC_extr_	1.43 ± 0.02	6 ± 0.08	2.53 ± 0.05	11 ± 0.23
IL_crr_^a^	– ^b^	– ^b^	0.70 ± 0.02	3 ± 0.08
MWL	1.79 ± 0.07	8 ± 0.28	2.64 ± 0.07	11 ± 0.30
ILL	0.24 ± 0.04	1 ± 0.15	0.51 ± 0.01	2 ± 0.05
EOL	0.23 ± 0.04	1 ± 0.18	0.18 ± 0.01	1 ± 0.01
Reference samples				
Cellulose acetate	10.57 ± 0.54	45 ± 2.33	9.77 ± 0.02	42 ± 0.08
Ethyl acetate	11.04 ± 0.18	48 ± 0.76	10.94 ± 0.08	47 ± 0.36
Beech wood	1.15 ± 0.04	5 ± 0.17	1.47 ± 0.05	6 ± 0.22
Beech wood acetylated	6.45 ± 0.05	28 ± 0.21	5.47 ± 0.03	24 ± 0.14
*Miscanthus x giganteus*	0.97 ± 0.01	4 ± 0.05	1.61 ± 0.08	7 ± 0.36
Coconut trunk	1.01 ± 0.07	4 ± 0.28	1.69 ± 0.04	7 ± 0.15

^a^ Cellulose rich residue from [EMIm][OAc] treatment; ^b^ not determined by this method because there was no sample left.

## Supplementary Materials

The following are available online at http://www.mdpi.com/2073-4360/10/6/619/s1, Table S1. Raman Band assignment for in-situ TC; Table S2. 2D HSQC ^13^C-^1^H correlations signals and assignment for TC_extr_ and TC lignin Isolates; Tables S3. a–e. HSQC volume integrations for estimation of acetyl groups associated units; Figure S1. 1 H NMR Integrals from which values of aromatic and aliphatic acetate were estimated; Figure S2. Full spectra and volume integrations for the TC_extr_ and lignin samples; Plate S1. Samples after incubation and GC measurement for Zemplén Transesterification analysis with EOL depicting higher solubility compared to MWL and ILL.

## References

[1] Ralph J. (2010). Hydroxycinnamates in lignification. Phytochem Rev..

[2] Del Rio J.C., Rencoret J., Prinsen P., Martinez A.T., Ralph J., Gutierrez A. (2012). Structural Characterization of Wheat Straw Lignin as Revealed by Analytical Pyrolysis, 2D-NMR, and Reductive Cleavage Methods. J. Agric. Food Chem..

[3] Vogel J. (2008). Unique aspects of the grass cell wall. Curr. Opin. Plant Biol..

[4] Del Río J.C., Marques G., Rencoret J., Martínez A.T., Gutiérrez A. (2007). Occurrence of naturally acetylated lignin units. J. Agric. Food Chem..

[5] Romero I., Moya M., Sanchez S., Ruiz E., Castro E., Bravo V. (2007). Ethanolic fermentation of phosphoric acid hydrolysates from olive tree pruning. Ind. Crops Prod..

[6] Zweckmair T., Becker M., Ahn K., Hettegger H., Kosma P., Rosenau T., Potthast A. (2014). A novel method to analyze the degree of acetylation in biopolymers. J. Chromatogr..

[7] Brandt A., Chen L., von Dongen B., Welton T., Hallet J.P. (2015). Structural changes in lignins isolated using an acidic ionic liquid water mixture. Green Chem..

[8] Zweckmair T., Hettegger H., Abushammala H., Bacher M., Potthast A., Laborie M.P., Rosenau T. (2015). On the mechanism of the unwanted acetylation of polysaccharides by 1,3-dialkylimidazolium acetate ionic liquids: Part 1—Analysis, acetylating agent, influence of water, and mechanistic considerations. Cellulose.

[9] Abushammala H., Hettegger H., Bacher M., Korntner P., Potthast A., Rosenau T., Laborie M.P. (2017). On the mechanism of the unwanted acetylation of polysaccharides by 1,3-dialkylimidazolium acetate ionic liquids: Part 2—The impact of lignin on the kinetics of cellulose acetylation. Cellulose.

[10] Cetinko O.P., Dibble D.C., Cheng G., Kent M.S., Knierim B., Auer M., Wemmer D.E., Pelton J.G., Melnichenko Y.B., Ralph J. (2010). Understanding the Impact of Ionic Liquid Pretreatment on Eucalyptus. Biofuels.

[11] Abushammala H., Pontes J.F., Gomes G.H., Osorio-Madrazo A., Thire R.M.S.M., Pereiral V.F., Laborie M.P. (2015). Swelling, Viscoelastic, and Anatomical Studies on Ionic Liquid-Swollen Norway Spruce as a Screening Tool toward Ionosolv Pulping. Holzforschung.

[12] Abushammala H., Krossing I., Laborie M.P. (2015). Ionic Liquid-Mediated Technology to Produce Cellulose Nanocrystals Directly from Wood. Carbohydr. Polym..

[13] Abushammala H., Goldsztayn R., Leao A., Laborie M.P. (2016). Combining Steam Explosion with 1-Ethyl-3-Methylimidazlium Acetate Treatment of Wood Yields Lignin-Coated Cellulose Nanocrystals of High Aspect Ratio. Cellulose.

[14] Voigt W., Porte H. (2007). South Africa National Biodiversity Institute. http://www.plantzafrica.com/planttuv/typhacapen.htm.

[15] Junrungreang S., Jutvapornvanit P. (1996). Possibility of Cattail for Waste-Water Treatment.

[16] Audu I.G., Ziegler-Devin I., Winter H., Bremer M., Hoffmann A., Fischer S., Laborie M.-P., Brosse N. (2017). Impact of Ionic Liquid 1-Ethyl-3-Methylimidazolium Acetate Mediated Extraction on Lignin Features. Green Sustain. Chem..

[17] Ralph J., Lu F. (1998). The DFRC Method for Lignin Analysis. 6. A Simple Modification for Identifying Natural Acetates on Lignins. J. Agric. Food Chem..

[18] Del Rio J.C., Prinsen P., Rencoret J., Nieto L., Jimenez-Barbero J., Ralph J., Martinez A.T., Gutierrez A. (2012). Structural characterization of lignin in the Cortex and pith of Elephant grass (Pennisetum Purpureum) stems. J. Agric. Food Chem..

[19] Lu F., Ralph J., Lu F. (2014). The DFRC (Derivatization followed by reductive cleavage) method and its applications for lignin characterization. Lignin.

[20] Schafer J., Urbat F., Rund K., Bunzel M.J. (2015). A Stable-Isotope Dilution GC-MS Approach for the Analysis of DFRC (Derivatization Followed by Reductive Cleavage) Monomers from Low Lignin Plant Materials. J. Agric. Food Chem..

[21] Wurzburg O.B. (1964). Methods in Carbohydrate Chemistry.

[22] Levigne S., Thomas M., Ralet M.C., Quemener B., Thibault T.F. (2002). Determination of the degrees of methylation and acetylation of pectins using a C18 column and internal standards. Food Hydrocoll..

[23] Hames B., Ruiz R., Scarlata C., Sluiter A., Sluiter J., Templeton D. (2008). Preparation of Samples for Compositional Analysis. Laboratory Analytical Procedure (LAP), National Renewable Energy Laboratory.

[24] Obst J.R., Kirk T.K. (1988). Isolation of Lignin. Methods Enzymol..

[25] Rencoret J., Marques G., Gutierrez A., Ibarra D., Li J., Gellerstedt G., Santos J.I., Jimene-Barbero J., Martinez A.T., del Rio J.C. (2008). Structural Characterization of Milled Wood Lignins from Different Eucalypt Species. Holzforschung.

[26] Sun N., Rahman M., Qin Y., Maxim M.L., Rodriguez H., Rogers R.D. (2009). Complete Dissolution and Partial Delignification of Wood in Ionic Liquid 1-Ethyl-3-Methylimidazolium Acetate. Green Chem..

[27] El Hage R., Brosse N., Sannigrahi P., Ragauskas A. (2010). Effects of Process Severity on the Chemical Structure of Miscanthus Ethanol Organosolv Lignin. Polym. Degrad. Stab..

[28] Lu F.C., Ralph J. (2003). Non-degradative dissolution and acetylation of balled milled plant cell walls high-resolution solution state NMR. Plant J..

[29] Rencoret J., Marques G., Gutierrez A., Nieto L., Santos J.I., Jimene-Barbero J., Martinex A.T., del Rio J.C. (2009). HSQC-NMR analysis of lignin in woody (Eucalyptus globulus and Picea abies) and non-woody (Agave sisalana) ball milled and plant materials at the gel state. Holzforchung.

[30] Guiochon G., Guillemin C.L. (1988). Quantitative Analysis by Gas Chromatography: Measurement of Peak Area and Derivation of Sample Composition. Laboratory Analyses and On-Line Process Control.

[31] Kim D.-Y., Nishiyama Y., Kuga S. (2002). Surface acetylation of bacterial cellulose. Cellulose.

[32] Agarwal U.P., Ralph S.A. (1997). FT-Raman Spectroscopy of Wood: Identifying Contributions of Lignin and Carbohydrate Polymers in the Spectrum of Black Spruce (Picea mariana). Appl. Spectrosc..

[33] Lupoi J.S., Smith E.A. (2011). Characterization of Woody and Herbaceous Biomasses Lignin Composition with 1064 nm Dispersive Multichannel Raman Spectroscopy. Appl. Spectrosc..

[34] Willey J.H., Atalla R.H. (1987). Band assignments in the Raman spectra of celluloses. Carbohydr. Res..

[35] Saariaho A., Jaaskelainen A., Nuopponen M., Vuorinen T. (2003). Ultra Violet Raman Spectroscopy in lignin analysis: Determination of characteristic vibrations of pHydroxophenyl, Guaiacyl, and Syringyl lignin structures. Appl. Spectrosc..

[36] Meyer M.W., Lupoi J.S., Smith E.A. (2011). 1064 nm dispersive multichannel Raman spectroscopy for the analysis of plant lignin. Anal. Chim. Acta.

[37] Lin S.Y., Dence C.W., Lundquist (1992). Proton (1H) NMR spectroscopy. Methods in Lignin Chemistry.

[38] Li S., Lundquist L. (1994). A new method for the analysis of phenolic groups in lignin by 1H NMR spectroscopy. Nordic Pulp Pap. Res. J..

[39] Mansfield S.D., Kim H., Lu F., Ralph J. (2012). Whole plant cell wall characterization using solution-state 2D NMR. Nat. Protoc..

[40] Meng L., Kang S., Zhang X., Wu Y., Sun R. (2012). Comparative Characterization of Lignins Extracted from Cotton Stalk Based on Complete Dissolution in Different Systems. Ind. Eng. Chem. Res..

[41] Kim H., Ralph J., Akiyama T. (2008). Solution-state 2D NMR of Ball-milled Plant Cell Wall Gels in DMSO-*d*_6_. Bioenergy Res..

[42] Ralph J., Lundquist K., Brunow G., Lu F., Kim H., Schatz P.F., Marita J.M., Hatfield R.D., Ralph S.A., Christensen J.H. (2004). Lignins: Natural polymers from oxidative coupling of 4-hydroxyphenyl-propanoids. Phytochem. Rev..

[43] Rencoret J., del Rio J.C., Gutierrez A., Martinez A.T., Li S., Parkas J., Lundquist K. (2011). Origin of the acetylated structures present in white birch (Betula pendula Roth) milled wood lignin. Wood Sci. Technol..

[44] Lapierre C., Rolando C. (1988). Thioacidolyses of pre-methylated lignin samples from Pine compression and Poplar woods. Holzforschung: Int. J. Biol. Chem. Phys. Technol. Wood..

[45] Bethge P.O., Lindströnt K. (1973). Determination of O-acetyl groups in wood. Svensk Papperstidning Nr.

[46] Mansson P., Samuelsson B. (1981). Quantitative Determination of O-Acetyl and Other O-Acyl Groups in Cellulosic Materials. Sven. Papperstidn..

[47] Balaban M., Ucar G. (2003). Estimation of volatile acids in wood and bark. Holz als Roh-und Werkst.

[48] Bacon J.S.D., Gordon A.H., Morris E.J. (1975). Acetyl Groups in Cell-Wall Preparations from Higher Plants. Biochem. J..

[49] Nabarlatz D., Ebringerová A., Montané D. (2007). Autohydrolysis of agricultural by-products for the production of XYLO-oligosaccharides. Carbohyd. Polym..

[50] Kumar R., Mago G., Balan V., Wyman C.E. (2009). Physical and chemical characterizations of corn stover and poplar solids resulting from leading pretreatment technologies. Bioresour. Technol..

[51] Rémond C., Aubry N., Crônier D., Noël S., Martel F., Roge B., Rakotoarivonina H., Debeire P., Chabbert B. (2010). Combination of ammonia and xylanase pretreatments: Impact on enzymatic xylan and cellulose recovery from wheat straw. Bioresour. Technol..

[52] Mante O.D., Babu S.P., Amidon T.E. (2014). A comprehensive study on relating cell-wall components of lignocellulosic biomass to oxygenated species formed during pyrolysis. J. Anal. Appl. Pyrolysis.

[53] Haffner F.B., Mitchell V.D., Arundale R.A., Bauer S. (2013). Compositional analysis of Miscanthus giganteus by near infrared spectroscopy. Cellulose.

[54] Nsaful F., Collard F., Carrier M., Görgens J.F., Knoetze J.H. (2015). Lignocellulose pyrolysis with condensable volatiles quantification by thermogravimetric analysis—Thermal desorption/gas chromatography–mass spectrometry method. J. Anal. Appl. Pyrolysis.

[55] Garrote G., Domínguez H., Parajó J.C. (2002). Autohydrolysis of corncob: Study of non-isothermal operation for xylooligosaccharide production. J. Food Eng..

[56] Fujii Y., Azuma J.I., Marchessault R.H., Morin F.G., Aibara S., Okamura K. (1993). Chemical composition change of bamboo accompanying its growth. Holzforschung.

[57] Gosselink R.J.A., van Dam J.G., Zomers F.H.A. (1995). Combined hplc analysis of organic acids and furans formed during organosolv pulping of fiber hemp. J. Wood Chem. Technol..

[58] De Carvalho D.M., Sevastyanova O., Penna L.S., da Silva B.P., Lindström M.E., Colodette J.L. (2015). Assessment of chemical transformations in eucalyptus, sugarcane bagasse and straw during hydrothermal, dilute acid, and alkaline pretreatments. Ind. Crops Prod..

[59] Iiyama K., Bach T.L.T., Natsuki K., Stone B.A. (1994). Rapid and simple determination of o-acetyl groups bound to plant cell walls by acid hydrolysis and 1h NMR measurement. Phytochemistry.

[60] Suzuki S., Rodriguez E.B., Iiyama K., Saito K., Shintani H. (1998). Compositional and structural characteristics of residual biomass from tropical plantations. J. Wood Sci..

[61] Thammasouk K., Tandjo D., Penner M.H. (1997). Influence of extractives on the analysis of herbaceous biomass. J. Agric. Food Chem..

[62] Gallacher J., Snape C.E., Hassan K., Jarvis M.C. (1994). Solid-state ^13^C NMR study of palm trunk cell walls. J. Food Agric..

[63] Beckers E.P.J., Bongers H.P.M., van der Zee M.E., Sander C. Acetyl content determination using different analytical techniques. Proceedings of the First European Conference on Wood Modification.

[64] Zhang G., Huang K., Jiang X., Huang D., Yang Y. (2013). Acetylation of rice straw for thermoplastic applications. Carbohyd. Polym..

[65] Puls J. (1997). Chemistry and biochemistry of hemicelluloses: Relationship between hemicellulose structure and enzymes required for hydrolysis. Macromol. Symp..

[66] Pinto P.C., Evtuguin D.V., Neto C.P. (2005). Structure of hardwood glucuronoxylans: Modifications and impact on pulp retention during wood Kraft pulping. Carbohyd. Polym..

