# Supplementary material for: Acetyl Groups in Typha capensis: Fate of Acetates during Organosolv and Ionosolv Pulping
Source: Polymers (Basel). 2018 Jun 5;10(6):619. doi: 10.3390/polym10060619 (PMC6404047; doi:10.3390/polym10060619)
Supplement: Supplementary file 1 [file polymers-10-00619-s001.pdf]

SUPPLEMENTARY INFORMATION

Acetyl groups in *Typha capensis*: Fate of acetates during organosolv and ionosolv pulping

Idi Guga Audu [1,2,3,4,*], Nicolas Brosse [2], Heiko Winter [1,3], Anton Hoffmann [4], Martina Bremer [4], Steffen Fischer [4] and Marie-Pierre Laborie [1,3]

[1] Chair of Forest Biomaterials, University of Freiburg, Werthmannstr. 6, 79085 Freiburg i. Br., Germany; idig.audu@gmail.com, heiko.winter@biomat.uni-freiburg.de, marie-pierre.laborie@biomat.uni-freiburg.de

[2] LERMAB, Faculté des Sciences et Technologies, University de Lorraine, Boulevard des Aiguillettes BP 70239, 54506 Vandœuvre lès Nancy Cedex, France; Nicolas.Brosse@univ-lorraine.fr

[3] Freiburg Materials Research Center (FMF), University of Freiburg, Stefan-Meier-Str. 21, 79104 Freiburg i. Br., Germany;

[4] Institute of Plant and Wood Chemistry, Technische Universität Dresden, Pienner Straße 19, 01737 Tharandt, Germany; anton.hoffmann@forst.tu-dresden.de, martina.bremer@forst.tu-dresden.de, sfischer@forst.tu-dresden.de

* Correspondence: idig.audu@gmail.com; Tel.: +49-152-148-040-56

Table S1. Raman Band assignment for in-situ TC

Band cm^{-1}	Intensity	Assignment
3068	0.54	C-H aromatic stretch
2938	8.32	C-H stretch in OCH_3, asymmetric, acetylated
1710	1.27	Carbonyl stretch, if broad could be protein or acyl group in lignin
1632	17.18	Double bond C=C Stretching vibration; Lignin C=C stretch of coniferaldehyde, sinapaldehyde, phenolic esters
1605	30.56	Aromatic C=C stretching vibrations
1464	4.52	lignin OCH_3 deformation, cellulose HCC and HOC bend
1380	2.35	Lignin symmetric CH deformation; cellulose HCC, HCO and HOC Bend
1332	1.45	Aliphatic O-H bend
1267	2.17	Aryl-O of aryl-OH and aryl-O-CH_3; guaiacyl ring (with C=O group) mode
1204	1.27	lignin O-CH_3 vibrations
1172	4.52	Lignin hydroxyl COH bend, aromatic skeletal vibrations
1122	3.26	Cellulose CC and CO stretch, lignin methoxy vibrations, aryl CH Bend
1095	3.62	Cellulose CC and CO stretch
1038	0.18	Cellulose CC and CO stretching; Lignin CH_3 wagging, CH_3 out-of-plane rock, aromatic skeletal vibrations, methoxy vibrations
982	1.09	Lignin CCH wag, aromatic skeletal vibrations
897	2.17	Cellulose HCC and HCO bending; Trans C-H wag
860	0.72	In phase C-C-O stretch
805	0.90	CO stretch; aryl symmetric CH bend, CH out of plane bend

Table S2. 2D HSQC ^{13}C-^{1}H correlations signals and assignment for TC_{extr} and TC lignin Isolates. Acetylated units in green highlights.

**TC_{extr}**	**MWL**	**ILL**	**EOL**		**Assignment**
	**δC/δH (ppm)**				
20.6/1.98	20.5/2.05				methyl in acetate peak linked to xylan moieties
	53.6/3.0	53.0/3.09		B_β	Cβ-Hβ in β-β', α-O- γ or γ-O-α linkages (resinol) substructures
	53.5/3.69		53.5/3.65	C_β	Cβ-Hβ in β-5' & α-O-4' phenylcoumaran substructures
55.1/3.68	55.1/3.67	54.9/3.7	*55.3/3.69*	MeO	C-H in methoxyls
	59.6/3.52	59.4/3.41 - 3.16	*60.4/3.3*	A_γ	CγHγ in β-O-4' substructures and others
60.3/3.45	59.6/3.16	59.1/3.18 & 2.79		D_β	Cβ-Hβ in β-1 Spirodienone) substructures formed by β-1', α-O-α' linkages
		61.9/4.04		J_γ	Cγ-Hγ in Cinnamyl alcohol end groups
62.5/3.8	62.5/4.21; 62.4/3.64	61.9/4.29-3.78	63.2/4.44	A_γ	CγHγ in γ-acetylated β-O-4' substructures
		63.1/3.69		B_γ	Cγ-Hγ in β-5 (phenolcoumaran) substructutures (resinols)
	64.1/4.71			J_γ	Cγ-Hγ in Cinnamyl acetate end groups
			66.5/4.08	HK	Hibbert keton
		70.4/4.13	*71.0/4.18*	B_β	Resinol substructure β-β' in Cγ/Hγ correlations
	71.4/4.1, 3.76	71.6/3.78	*71.1/4.31*	B_γ	Cγ-Hγ in β-β' , α-O-γ or γ-O-α linkages (resinol) substructures
71.3/4.78	71.2/4.78	71.6/4.87	71.6/4.83	A_α	Cα-Hα in β-O-4' substructures
			72.5/3.14	A_α	Cα-Hα in β-O-4' substructures
73.2/2.87	73.3/3.16	72.7/3.01		X2	C2−H2 in 2-O-acetyl-β-D-xylopyranoside
		73.7/3.26		X3	C3−H3 in β-D-xylopyranoside
74.5/4.73	74.5/4.73	74.3/3.86		X3	C3−H3 in 3-O-acetyl-β-D-xylopyranoside
	75.2/3.49	75.4/3.52		X4	C4−H4 in β-D-xylopyranoside
79.5/3.35				A_α	Cα-Hα in β-O-4' substructure (α, β-diaryl ethers)
81.6/3.79		81.6/4.9	80.8/4.56	D_α	Cα-Hα in β-1 (Spirodienone) substructures formed by β-1', α-O-α' linkages
83.2/4.23	83.1/4.24	83.3/4.29		$A_{\beta(G)}$	Cβ-Hβ in β-O-4' substructures linked to a G unit
83.3/3.73				A_α	Cα-Hα in 5-5' (dibenzodioxocin) substructures (β-O-4' linkages)
	84.7/4.58		*84.9/4.58*	$A_{\beta(G)}$	Cβ-Hβ in β-O-4' substructures linked to a G unit
85.7/3.4	85.4/4.05	84.7/4.65		B_α	Cα-Hα in β-β', α-O- γ or γ-O-α linkages (resinol) substructures
		86.1/4.05		$A_{\beta(S)}$	Cβ-Hβ in β-O-4' substructures linked to a S unit
	86.6/5.39	86.4/5.47	*86.7/5.42*	C_α	Cα-Hα in β-5' phenylcoumaran substructures formed by β-5' and α-O-4
98.4/4.84	98.6/4.66	97.6/4.91		β-D_m	β-D-Mannosyl (mannose residues)

<u>**TC$_{extr}$**</u>	<u>**MWL**</u>	<u>**ILL**</u>	<u>**EOL**</u>		<u>**Assignment**</u>
	**δC/δH (ppm)**				
99.0/4.60	99.4/4.45	100.6/4.45		α-D$_{ga}$	α-D-Galactosyl (galactose residues)
102.0/4.23	102.0/4.25	101.9/4.22		β-D$_{gl}$	β-D-Glucosyl (Glucose residues)
103.1/6.63	103.1/6.65	*103.8/6.77*	103.5/6.67	S	C2-H2 and C6-H6 in syringyl units
107.3/5.12	105.6/7.19	106.6/7.26	105.9/7.3	S'	C2-H2 and C6-H6 in oxidized (Cα=O) syringyl units
110.4/6.85	109.5/6.98	110.6/6.99	109.5/7.07	G	C2-H2 in guaiacyl units (G)
109.7/7.29	110.5/7.38	*109.6/7.25*	110.6/7.32	G'	C2-H2 in oxydized α-ketone structure of G'
	112.5/6.11	*112.4/6.11*	111.4/6.63	G	C2-H2 in guaiacyl units (G)
115.3/6.7		*115.1/6.76 & 6.92*	115.2/6.63	G/H	C3−H3 and C5−H5 in p-coumarate (PCA); overlaps of H & G
118.8/6.72	119.4/6.77	*118.9/6.84*	118.7/6.87 & 6.7	G	C6-H6 in guaiacyl units (G)
		119.9/7.66	119.20/7.16	G	C6-H6 in guaiacyl units (G)
	122.1/7.12-7.49			U	unknown
	127.4/7.21	*127.8/7.22 & 6.23,*	126.9/7.25	H	C2,6-H2,6 in p-hydroxyphenyl units (H)
		128.2/6.44		J	Cα-Hα in cinnamyl alcohol end-groups
129.0/5.27		129.4/5.3	129.6/5.24	F	Cα and C$_{\beta}$ of p-hydrocycinnamyl alcohol (F)
	128.3/7.42, 5.28	128.7/7.26	128.2/7.66	P	p-coumarate 2,6 corelations
129.7/7.45	130.5/7.5	*129.5/7.43*	129.9/7.46	P	C2−H2 and C6−H6 in p-coumarate (PCA)
	144.6/7.64-7.14		144.4/7.49	P	Cα−Hα in p-coumarate (PCA) and ferulate (FA)

Tables S3a – e. HSQC volume integrations for estimation of acetyl groups associated units.

Table S3a. Integral values for TC_{extr}						
Object	Integral [abs]	Integral [rel]	ν(F2) [ppm]	ν(F1) [ppm]	Normalization	% Integral
Integral 1	2910600000	1	2.30	38.35	277.78	66.99
Integral 2	69189000	0.0238	1.86	20.50	6.61	1.59
Integral 3	7521000	0.0026	0.80	12.98	0.72	0.17
Integral 4	27160000	0.0093	0.94	17.60	2.58	0.62
Integral 5	16555000	0.0057	0.83	21.58	1.58	0.38
Integral 6	61055000	0.021	1.32	23.73	5.83	1.41
Integral 7	207350000	0.0712	1.20	29.21	19.78	4.77
Integral 8	14292000	0.0049	1.89	25.99	1.36	0.33
Integral 9	4172000	0.0014	2.19	32.76	0.39	0.09
Integral 10	8769300	0.003	1.96	33.73	0.83	0.20
Integral 11	197820000	0.068	3.63	55.12	18.89	4.56
Integral 12	49656000	0.0171	4.12	62.00	4.75	1.15
Integral 13	254890000	0.0876	3.35	62.00	24.33	5.87
Integral 14	43323000	0.0149	3.75	81.35	4.14	1.00
Integral 15	143880000	0.0494	2.97	69.95	13.72	3.31
Integral 16	60852000	0.0209	3.32	77.26	5.81	1.40
Integral 17	4894000	0.0017	3.39	85.22	0.47	0.11
Integral 18	39267000	0.0135	4.72	72.00	3.75	0.90
Integral 19	8018700	0.0028	5.25	128.65	0.78	0.19
Integral 20	5790700	0.002	4.98	98.55	0.56	0.13
Integral 21	27192000	0.0093	4.55	98.66	2.58	0.62
Integral 22	69627000	0.0239	4.29	102.20	6.64	1.60
Integral 23	17105000	0.0059	6.59	103.60	1.64	0.40
Integral 24	10559000	0.0036	6.89	110.05	1.00	0.24
Integral 25	48226000	0.0166	6.60	113.92	4.61	1.11
Integral 26	8572100	0.0029	6.73	117.90	0.81	0.19
Integral 27	28264000	0.0097	7.12	127.57	2.69	0.65
		1.4927	97.542	1822.4254	414.6388889	100
					Ac in Lignin	7.92
					Ac in HS	4.90
					Ac Total	12.82

Table S3b. HSQC Integral values for MWL

Object	Integral [abs]	Integral [rel]	ν(F2) [ppm]	ν(F1) [ppm]	Normalization	% Integral
Integral 1	5402000000	1	3.61	53.46	4.56	32.71
Integral 2	999880000	0.1851	4.68	70.98	0.84	6.05
Integral 3	566290000	0.1048	1.84	19.27	0.48	3.43
Integral 4	676500000	0.1252	4.25	62.17	0.57	4.09
Integral 5	2174000000	0.4024	3.38	61.20	1.84	13.16
Integral 6	288290000	0.0534	2.99	70.23	0.24	1.75
Integral 7	331570000	0.0614	3.57	75.07	0.28	2.01
Integral 8	147310000	0.0273	4.08	69.69	0.12	0.89
Integral 9	236980000	0.0439	3.59	68.08	0.20	1.44
Integral 10	166390000	0.0308	4.94	81.19	0.14	1.01
Integral 11	301360000	0.0558	4.49	80.98	0.25	1.82
Integral 12	482880000	0.0894	4.03	84.10	0.41	2.92
Integral 13	159370000	0.0295	5.45	86.25	0.13	0.96
Integral 14	116420000	0.0216	4.24	100.87	0.10	0.71
Integral 15	942360000	0.1744	6.67	103.55	0.80	5.70
Integral 16	63524000	0.0118	7.22	104.52	0.05	0.39
Integral 17	1183700000	0.2191	7.04	110.97	1.00	7.17
Integral 18	596270000	0.1104	6.58	113.34	0.50	3.61
Integral 19	670470000	0.1241	6.75	118.50	0.57	4.06
Integral 20	160610000	0.0297	7.12	127.10	0.14	0.97
Integral 21	186560000	0.0345	7.46	128.82	0.16	1.13
Integral 22	64399000	0.0119	7.48	143.97	0.05	0.39
Integral 23	28805000	0.0053	6.19	127.85	0.02	0.17
Integral 24	23104000	0.0043	6.41	126.99	0.02	0.14
Integral 25	15588000	0.0029	8.54	148.81	0.01	0.09
Integral 26	41983000	0.0078	2.97	51.95	0.04	0.26
Integral 27	332640000	0.0616	1.15	27.77	0.28	2.01
Integral 28	60349000	0.0112	1.39	23.36	0.05	0.37
Integral 29	35910000	0.0066	7.14	121.94	0.03	0.22
Integral 30	23733000	0.0044	7.35	123.01	0.02	0.14
Integral 31	37650000	0.007	4.62	63.24	0.03	0.23
					13.96	100.00
					Ac in Lignin	20.41
					Ac in HS	7.18
					Ac Total	27.59

Table S3c. HSQC Integral values for ILL						
Object	Integral [abs]	Integral [rel]	ν(F2) [ppm]	ν(F1) [ppm]	Normalization	% Integral
Integral 1	3763800000	1	3.64	55.48	14.20	37.46
Integral 2	87731000	0.0233	1.18	14.85	0.33	0.87
Integral 3	66739000	0.0177	0.78	13.56	0.25	0.66
Integral 4	122960000	0.0327	0.77	19.15	0.46	1.22
Integral 5	136330000	0.0362	0.77	21.84	0.51	1.36
Integral 6	942360000	0.2504	1.16	28.72	3.56	9.38
Integral 7	21189000	0.0056	1.21	21.19	0.08	0.21
Integral 8	477890000	0.127	1.56	23.77	1.80	4.76
Integral 9	52131000	0.0139	1.94	26.24	0.20	0.52
Integral 10	2833900	0.0008	3.73	33.98	0.01	0.03
Integral 11	448810000	0.1192	4.80	71.18	1.69	4.46
Integral 12	409840000	0.1089	3.42	59.03	1.55	4.08
Integral 13	331290000	0.088	3.76	63.12	1.25	3.30
Integral 14	133770000	0.0355	3.15	63.22	0.50	1.33
Integral 15	227050000	0.0603	2.99	72.15	0.86	2.26
Integral 16	228270000	0.0606	3.28	72.68	0.86	2.27
Integral 17	216320000	0.0575	3.46	75.05	0.82	2.15
Integral 18	73794000	0.0196	3.79	70.86	0.28	0.73
Integral 19	240760000	0.064	3.98	86.12	0.91	2.40
Integral 20	95429000	0.0254	4.25	82.57	0.36	0.95
Integral 21	198790000	0.0528	4.23	101.82	0.75	1.98
Integral 22	37551000	0.01	5.42	87.09	0.14	0.37
Integral 23	671390000	0.1784	6.68	103.43	2.53	6.68
Integral 24	265010000	0.0704	6.99	110.52	1.00	2.64
Integral 25	293410000	0.078	6.76	113.86	1.11	2.92
Integral 26	131920000	0.035	6.80	118.48	0.50	1.31
Integral 27	40124000	0.0107	6.11	113.86	0.15	0.40
Integral 28	156650000	0.0416	7.17	128.37	0.59	1.56
Integral 29	50926000	0.0135	7.39	130.41	0.19	0.51
Integral 30	36345000	0.0097	5.27	129.87	0.14	0.36
Integral 31	31435000	0.0084	1.97	36.13	0.12	0.31
Integral 32	55035000	0.0146	4.07	61.72	0.21	0.55
					37.92	100.00
					Ac in Lignin	5.58
					Ac in HS	4.41
					Ac Total	9.99

Table S3d. HSQC Integral values for EOL						
Object	Integral [abs]	Integral [rel]	ν(F2) [ppm]	ν(F1) [ppm]	Normalization	% Integral
Integral 1	3381000000	1	3.58	55.20	2.94	24.46
Integral 2	566860000	0.1677	0.98	13.39	0.49	4.10
Integral 3	86442000	0.0256	0.79	21.56	0.08	0.63
Integral 4	71489000	0.0211	0.75	18.44	0.06	0.52
Integral 5	299070000	0.0885	1.40	24.03	0.26	2.16
Integral 6	198410000	0.0587	1.18	22.42	0.17	1.44
Integral 7	2070900000	0.6125	1.11	28.54	1.80	14.98
Integral 8	199170000	0.0589	1.33	31.88	0.17	1.44
Integral 9	74404000	0.022	1.91	25.86	0.06	0.54
Integral 10	71035000	0.021	2.01	29.19	0.06	0.51
Integral 11	310880000	0.0919	2.16	33.17	0.27	2.25
Integral 12	2201300000	0.6511	3.59	59.93	1.92	15.92
Integral 13	255830000	0.0757	3.37	64.23	0.22	1.85
Integral 14	164080000	0.0485	4.00	65.63	0.14	1.19
Integral 15	77999000	0.0231	5.26	129.05	0.07	0.56
Integral 16	37547000	0.0111	3.14	72.30	0.03	0.27
Integral 17	19500000	0.0058	4.44	62.73	0.02	0.14
Integral 18	428990000	0.1269	6.58	102.93	0.37	3.10
Integral 19	950070000	0.281	6.51	108.84	0.83	6.87
Integral 20	1148900000	0.3398	6.58	114.33	1.00	8.31
Integral 21	106860000	0.0316	7.18	106.69	0.09	0.77
Integral 22	143670000	0.0425	7.23	110.24	0.13	1.04
Integral 23	290070000	0.0858	6.69	119.16	0.25	2.10
Integral 24	529240000	0.1565	7.06	127.76	0.46	3.83
Integral 25	101390000	0.03	7.42	129.70	0.09	0.73
Integral 26	39421000	0.0117	7.42	123.57	0.03	0.29
					12.03	100.00
					Ac in Lignin	1.33
					Ac in HS	0.27
					Ac Total	1.60

Table S3e. Summary of acetate values as % of total integrals of the HSQC chemical shifts

Sample	Acetate on Lignin Moities (%)	Acetate on Xylan Moieties (%)	Total Acetate (%)
TC_{extr}	7.92	4.9	12.82
MWL	20.41	7.18	27.59
ILL	5.58	4.41	9.99
EOL	1.33	0.27	1.6

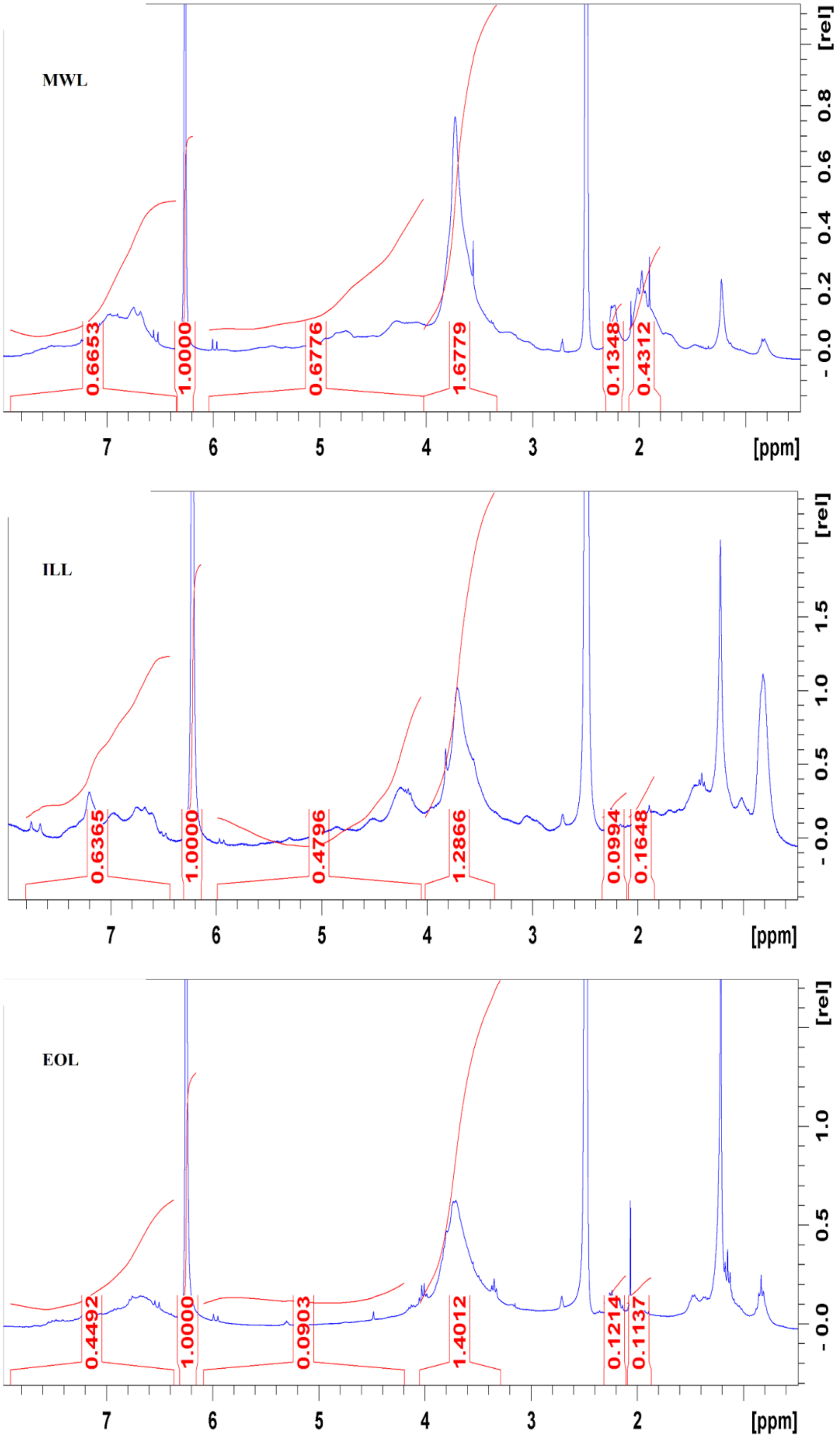

Figure S1. 1 H NMR Integrals from which values of aromatic and aliphatic acetate were estimated.

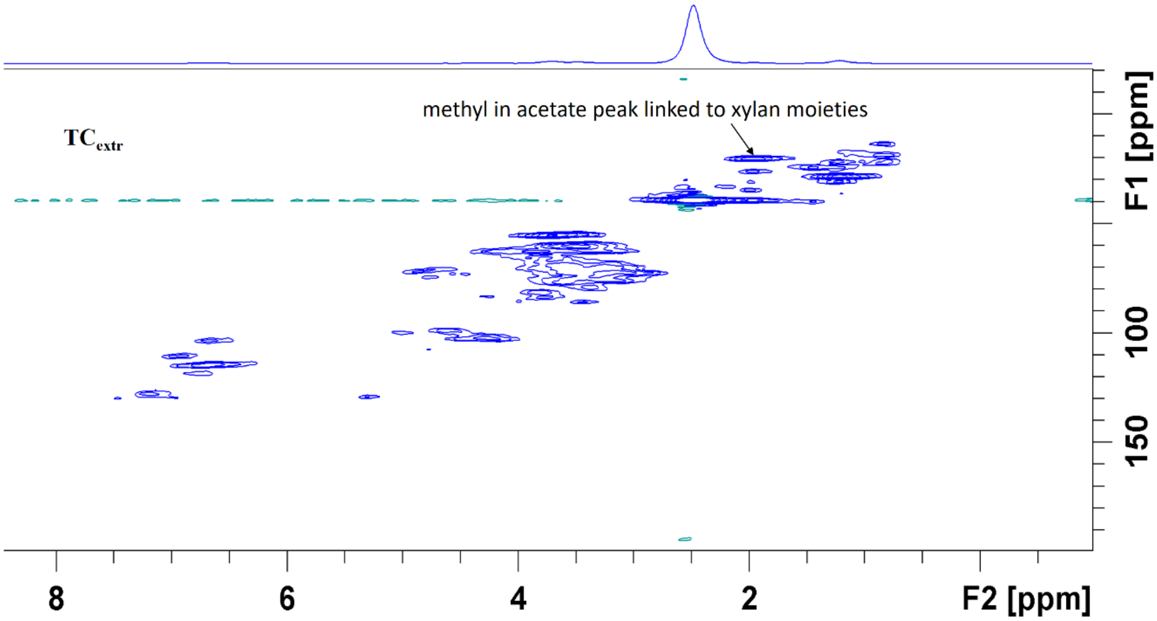

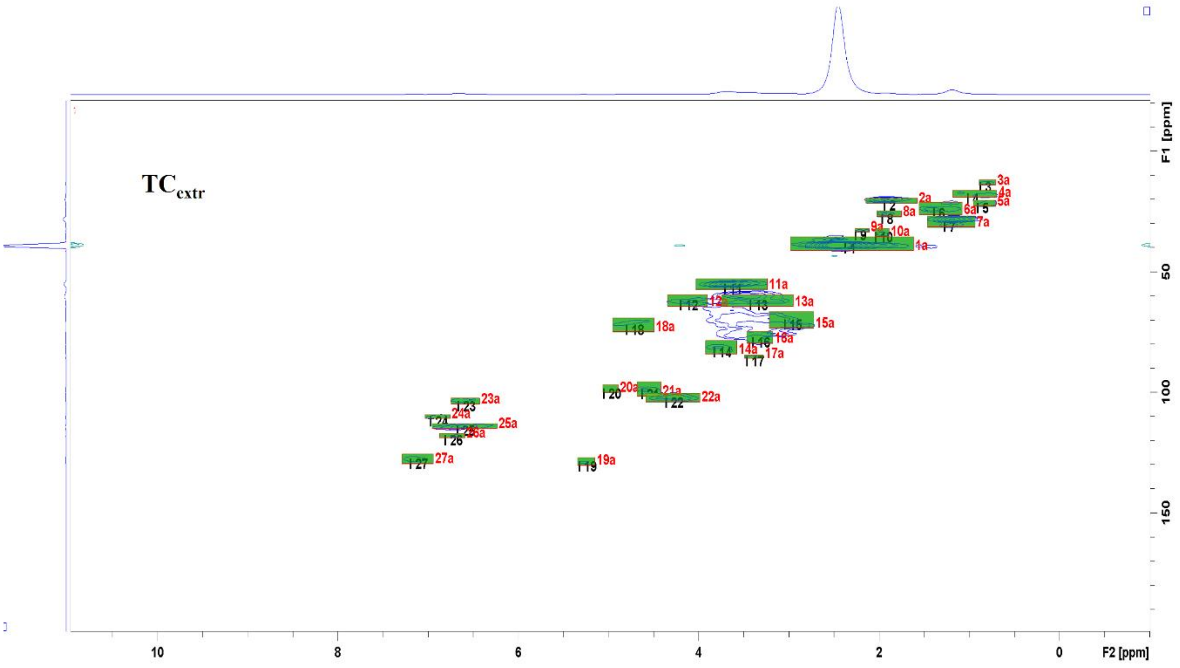

Figure S2a. Full spectra and volume integrations for TC_{extr} and lignin samples. NB. Methyl acetate linked to xylan are seen in the full spectrum.

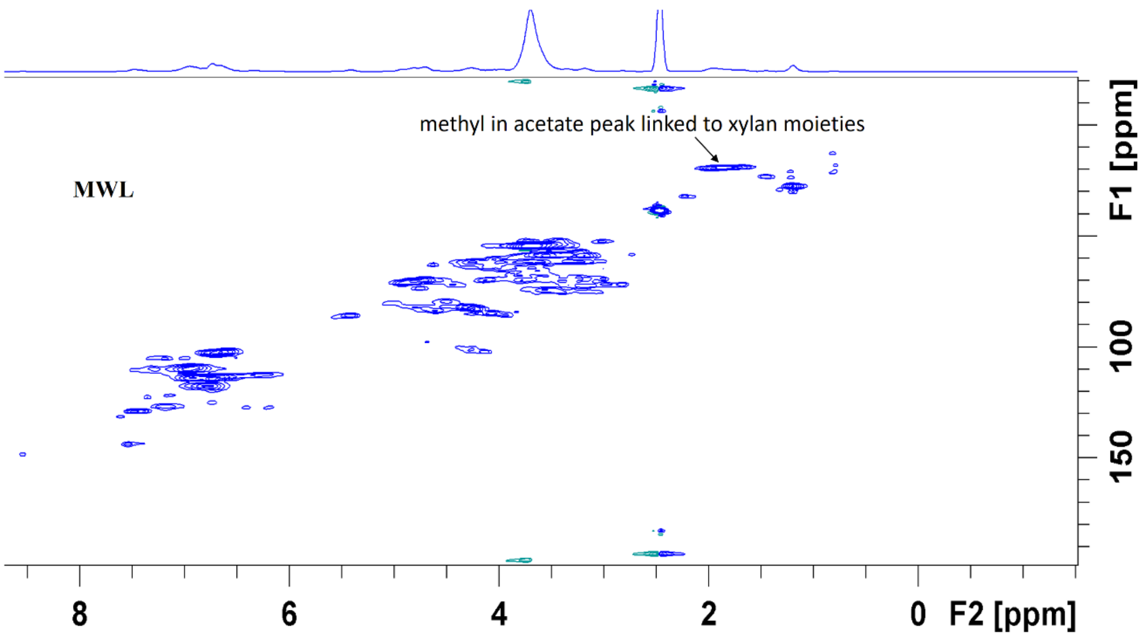

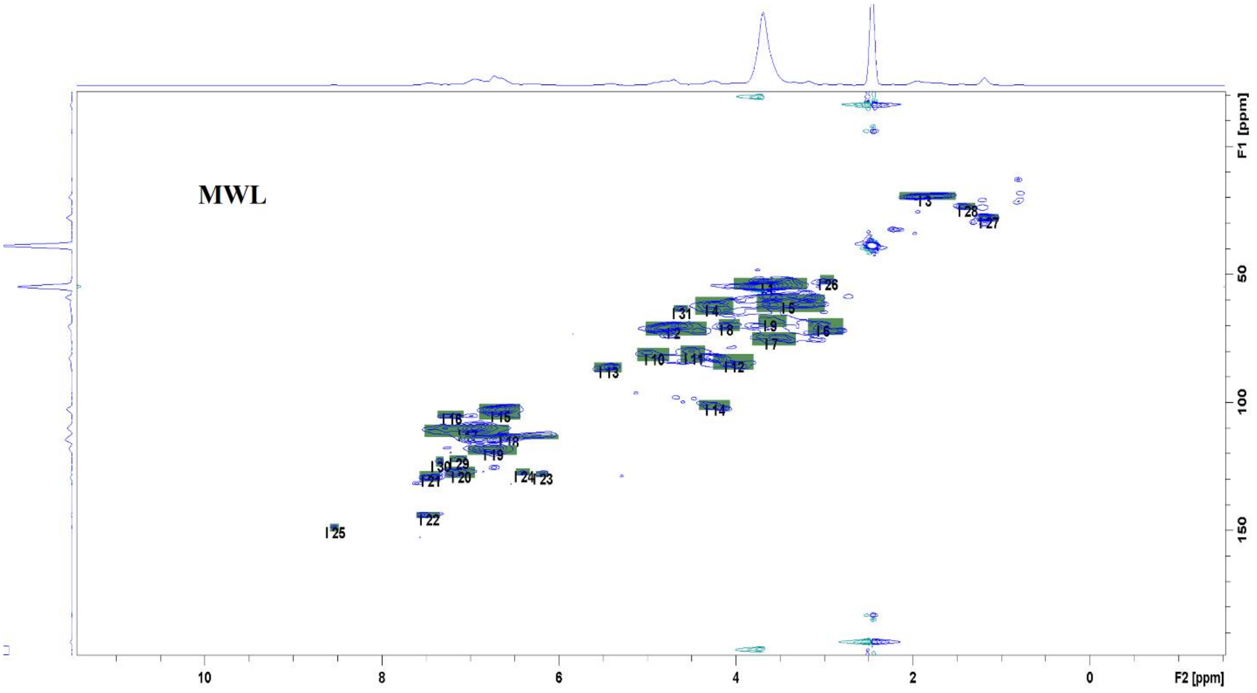

Figure S2b. Full spectra and volume integrations for TC MWL. NB. Methyl acetate linked to xylan are seen in the full spectrum.

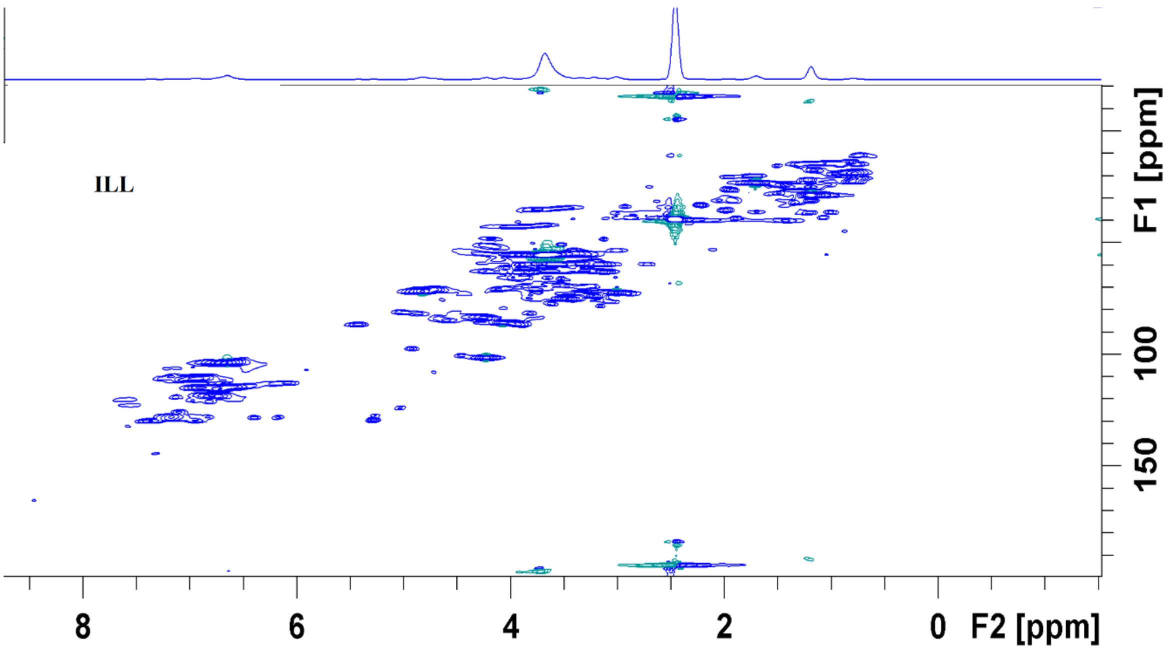

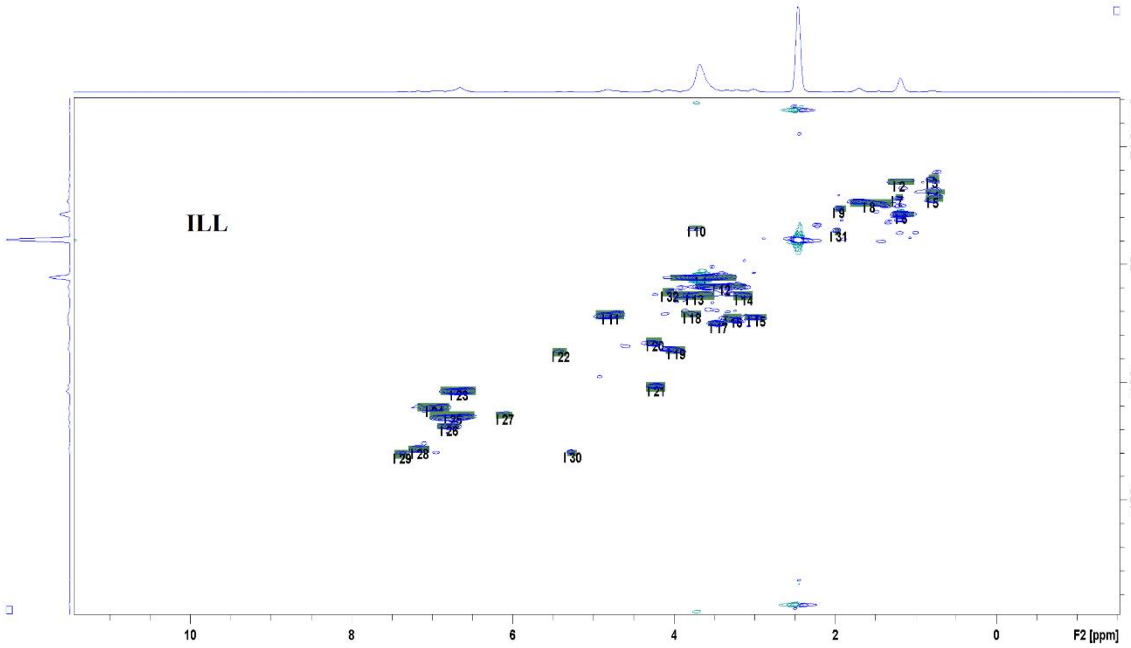

Figure S2c. Full spectra and volume integrations for TC ILL.

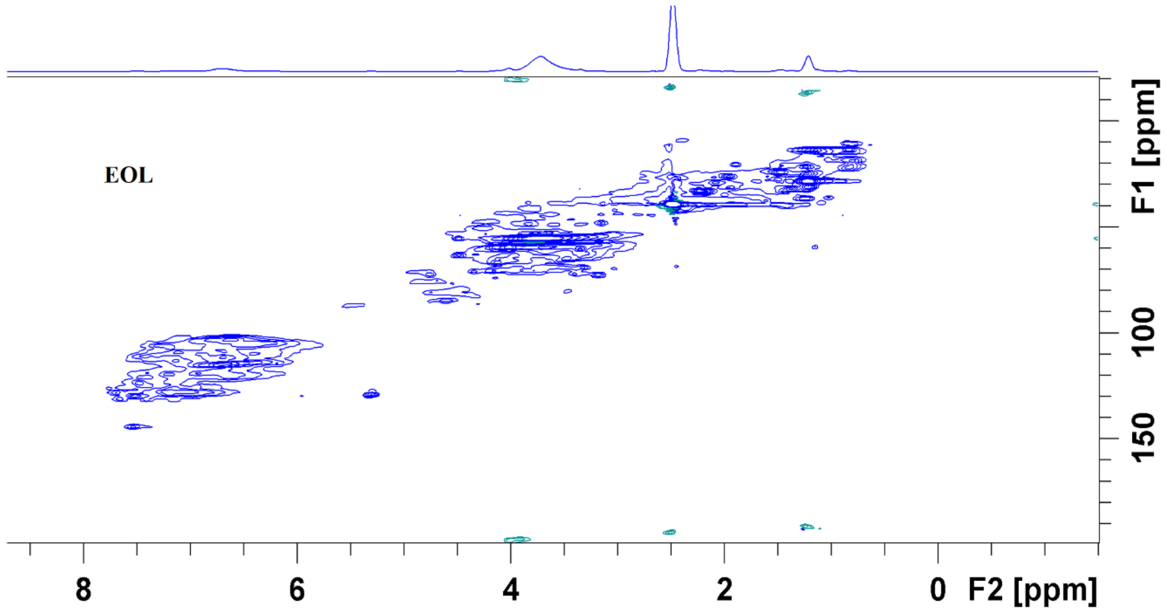

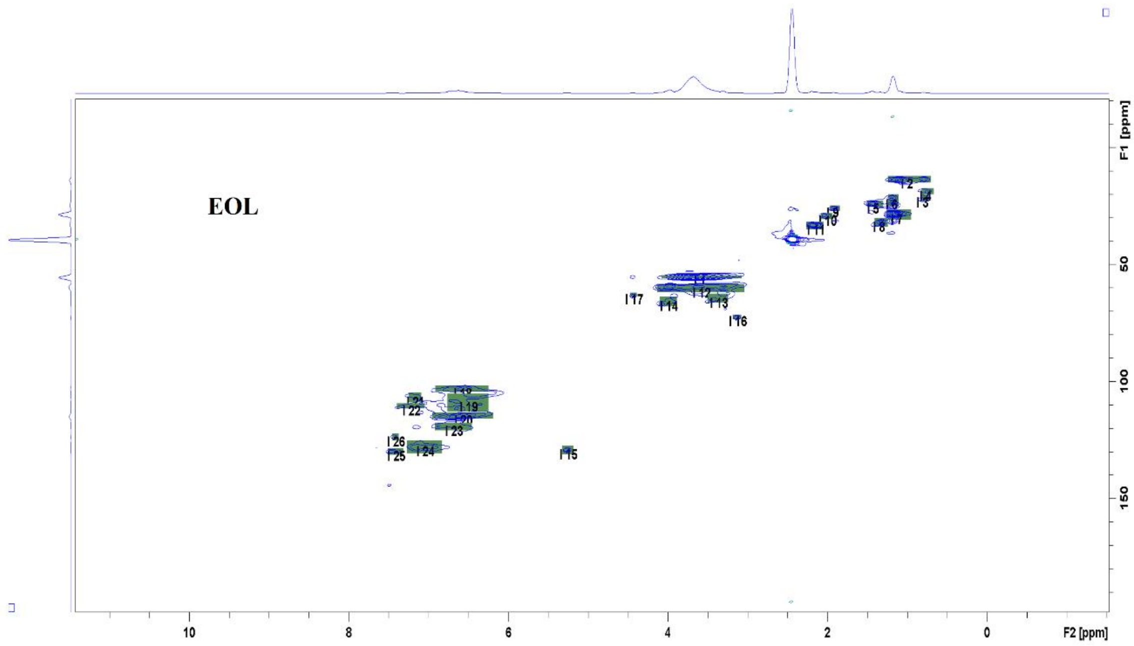

Figure S2d. Full spectra and volume integrations for TC EOL.

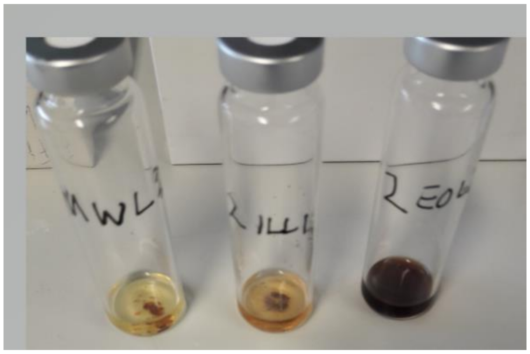

Plate S1. Samples after incubation and GC measurement for Zemplén Transesterification analysis with EOL depicting higher solubility compared to MWL and ILL.
